# Lipid accumulation product (LAP) was independently associatedwith obstructive sleep apnea in patients with type 2 diabetes mellitus

**DOI:** 10.1186/s12902-020-00661-x

**Published:** 2020-12-09

**Authors:** Lianqin Dong, Mingzhu Lin, Wengui Wang, Danyan Ma, Yun Chen, Weijuan Su, Zheng Chen, Shunhua Wang, Xuejun Li, Zhibin Li, Changqin Liu

**Affiliations:** 1 The School of Clinical Medicine, Fujian Medical University, Fuzhou, 350000 China; 2 Department of Endocrinology and Diabetes, The First Affiliated Hospital of Xiamen University, No.55 Zhenhai Road, Xaimen, 361003 China; 3 Xiamen Clinical Medical Center for Endocrine and Metabolic Diseases, Fujian Province Key Laboratory of Diabetes Translational Medicine, Xiamen, China; 4 School of Medicine, Xiamen University, Xiamen, China; 5 Epidemiology Research Unit, The First Affiliated Hospital of Xiamen University, No.55 Zhenhai Road, Xiamen, 361003 China

**Keywords:** Obstructive sleep apnea, Lipid accumulation product, Apnea-hypopnea index, Type 2 diabetes mellitus

## Abstract

**Background:**

Lipid accumulation product (LAP) is a new index based on a combination of waist circumference (WC) and serum triglycerides (TG) reflecting lipid accumulation. In this cross-sectional study, we aimed to explore whether LAP was independently associated with obstructive sleep apnea (OSA) in Type 2 diabetes mellitus (T2DM) patients.

**Methods:**

A cross-sectional study of 317 T2DM patients who underwent overnight polysomnography (PSG) tests was conducted. The clinical data between non-OSA group and OSA group were compared. Multivariable linear regression and multivariable logistic regression analyses were performed to determine associations of LAP, with apnea-hypopnea index (AHI) and OSA.

**Results:**

Among 317 patients, 219 (69.1%) were men, and the mean ages (±SD) were 51.4 (±13.5) years for men and 54.6 (±15.1) years for women (*p* = 0.067). The prevalence rates of OSA were 63.0% for men and 68.4% for women (*p* = 0.357). LAP (log-transformed) was significantly correlated with AHI (log-transformed), with the Pearson’s correlation coefficient of 0.170 (*p* = 0.002). With adjustment for potential confounding factors, multivariate linear regression analyses showed the association of LAP with AHI was not statistically significant, with the adjusted linear regression coefficients (95% CI) of per SD increase of LAP for AHI (log-transformed) was 0.092 (− 0.011–0.194, *p* = 0.080). Multivariate logistic regression analyses showed LAP was significantly associated with increased risk of OSA, with the adjusted OR (95%CI) of per SD increase of LAP of 1.639 (1.032–2.604, *p* = 0.036). However, as constituents of LAP, neither TG nor WC was significantly associated with AHI and OSA.

**Conclusion:**

LAP was independently associated with OSA and might be used as a potential OSA risk marker in T2DM patients, beyond the general index of obesity.

## Introduction

Obstructive sleep apnea (OSA) has gradually become a common sleep-disordered breathing in today’s society [1, 2]. OSA is characterized by the presence of repeated episodes of obstructive apnea and hypopnea during sleep [3]. Recent studied reported that the prevalence of moderate to severe OSA was 23.4% in women and 49.7% in men in the general population [4]. Patients with OSA often feel exhausted and sleepy during the daytime, which would result in the impairment in vigilance, concentration, cognitive function, social interactions and quality of life [5]. OSA usually develops in population who are overweight or obesity and is associated with hypertension and metabolic diseases [6]. Obesity is a prominent risk factor of OSA and increased visceral fat plays a role in the development of OSA [7], and weight control can effectively reduce the incidence of OSA in a longitudinal study [8]. In turns, sleep fragmentation and sleep restriction have been associated with increased food intake and consequent weight gain [9].

Type 2 diabetes mellitus (T2DM) is also a common disease around the world. Studies have shown that the prevalence rate of OSA is from 50 to 70% in diabetic patients [10]. Other studies also indicated a higher incidence rate of diabetes or insulin resistance in OSA patients [11]. OSA may induce diabetes through specific mechanisms such as glucose intolerance [12–14]. Data from murine models and human studies indicated that intermittent hypoxia and sleep fragmentation could result in the alternation of insulin sensitivity and glucose disposal [14]. Mok et al. showed that more severe OSA was associated with the poorer glycaemic control in T2DM patients expressed by the glycated hemoglobin A1c (HbA1c) levels after adjustment for age, race, sex, body mass index (BMI) and the duration of diabetes mellitus [11]. According to a study conducted by Nagayoshi and colleagues, they found that subjects with severe OSA were at greater risk of incident diabetes compared to persons classified as normal and independent of adiposity [14]. OSA has established a link with obesity and diabetes by the above reasons [9].

Rising BMI, waist circumference (WC), and neck circumference (NC) are important risk factors for the development of OSA [15]. Lipid accumulation product (LAP) is a new index based on a combination of WC and serum triglycerides (TG) reflecting lipid accumulation, which has been used as a marker of obesity [16]. A study included 2524 non-diabetic patients from China suggested that LAP could better identify insulin resistance than BMI and WC [17]. In a cohort study included 4508 non-diabetic participants with a median age of 42 years, higher LAP trajectories were associated with an increased risk of diabetes [18]. In a 6-year follow-up prospective study, the researchers found that LAP was superior to BMI in predicting diabetes in young individuals [19]. In T2DM patients, LAP was a useful tool to predict the risk for chronic diabetic complications, such as diabetic retinopathy [20]. However, in a large cross-sectional study with a total of 5539 subjects, Zou and colleagues also found LAP was of moderate efficiency in screening for OSA, which indicate that LAP is a simple and practical screening tool for OSA [21].

Epidemiological evidence indicate obesity could result in OSA via several mechanism including the collapsibility of the pharyngeal airway through excessive fat deposition [22], oxidative stress, inflammation, and even mutual organ interactions among the respiratory system, adipose tissue and intestine [23]. Similarly, OSA and T2DM share mutual risk factors such as obesity and insulin resistance [11]. However, there are few studies focusing on association between separate LAP and OSA prevalence in T2DM patients. Therefore, the aim of the present study was to investigate whether LAP is independently associated with the presence of OSA in T2DM.

## Methods

### Study subjects

During the period from 2013 to 2017, a total of 346 T2DM patients underwent overnight polysomnography (PSG) test had been recruited. Exclusion criteria were described as our previous study [24]. Of 346 patients, 317 patients who had complete data on clinical, PSG, and LAP were left for the present analysis. This study was approved by the Human Research Ethics Committee of the First Affiliated Hospital of Xiamen University (Xiamen, China).

### Demographic and biochemical measurements

Demographic data including body weight, standing height, WC, Hip circumference (HC), and arterial blood pressure were collected as described as our previous report [24]. HbA1c, TG, Total cholesterol (TC), HDL-cholesterol (HDL-c), and LDL-cholesterol (LDL-c) measurements have also been detailed described in the previous study [24]. BMI was calculated as follows: Body weight [kilograms] / (height [m])^2^. LAP was calculated as follows: (WC [cm] - 65) × (TG concentration [mmol/L]) for men, and (WC [cm] - 58) × (TG concentration [mmol/L]) for women, respectively [16].

### Polysomnography assessments

Polysomnography (PSG) (PSG, Comp medics, Abbotsford, Australia) was recorded as same as described in the previous report [24] and was scored according to generally accepted scoring methods [25]. The apnea-hypopnea index (AHI) was defined as the total number of obstructive apnea and hypopnea per hour of sleep. OSA was defined as AHI ≥ 5 [5].

### Statistical analyses

Data was presented as the mean ± standard deviation or as median (inter-quartile range, IQR) for continuous variable or number and percentage for categorical variable. Normality of all continuous variables were conducted. AHI and LAP did not follow normal distributions and therefore log-transformation on AHI and LAP were performed. Differences between OSA and non-OSA groups were analyzed on continuous variables using Student’s t test (t-test) for those with normal distribution and the Mann-Whitney U test for those with skewed distribution and on categorical variables using chi-square test. Pearson’s correlation analysis is used to analysis the correlation between LAP (log-transformed) and AHI (log-transformed).

Multivariable linear regression was used to explore the association of LAP with AHI (log-transformed). And multivariable logistic regression analysis was used to calculate the adjusted odds ratios (OR) and 95% confidence interval (CI) of LAP (per SD increase) for OSA in different models with adjustment for potential confounders. For both the multivariable linear regression and logistic regression analyses, no variables were adjusted for in model 1; age, sex, regular drinking, current smoking, T2DM duration, oral glucose-lowering agents (OGLA) and insulin use were adjusted for in model 2; systolic blood pressure (SBP), diastolic blood pressure (DBP), BMI, TC, LDL-c, and HbA1c were further adjusted for in model 3. To show if LAP was a better predictor than its two constitutes (TG and WC), additional regression analyses were further conducted for TG and WC separately and Akaike information criterions (AIC) were used to compare these consecutive models. A receiver operating characteristic (ROC) analysis was conducted for LAP to evaluate its ability to correctly detect moderate-severe OSA in T2DM patients. All *p*-values were two-sided and p-value< 0.05 was considered statistically significant. All statistical analyses were performed using SPSS version 21.0 software (IBM Corporation, Armonk, NY).

## Results

Among 317 patients with T2DM, 219 (69.1%) were men, and the mean ages (±SD) were 51.4 (±13.5) years for menand 54.6 (±15.1) years for women (*p* = 0.067). The prevalence rates of OSAwere 63.0% for men and 68.4% for women (*p* = 0.357).

### Demographic, lifestyle and clinical characteristics categorized by the severity of OSA

Table 1 showed the demographic, lifestyle and clinical characteristics between non-OSA group and OSA group. There were significant differences in ages (47.1 ± 14.8 vs. 55.3 ± 12.8, *p* < 0.001), statin use (71 (30.3%) vs. 163 (69.7%), *p* = 0.006), WC (95.3 ± 9.8 vs. 99.5 ± 9.6, *p* < 0.001), BMI (27.3 ± 4.2 vs. 28.2 ± 4.0, *p* = 0.039), HbA1c (10.1 ± 2.3 vs. 9.8 ± 2.2, *p* = 0.160), TG (median (IQR) 1.55 (1.15–2.20) vs. 1.76 (1.28–2.58), *p* = 0.041), LDL-c (3.3 ± 1.1 vs. 3.1 ± 1.0, *p* = 0.048), AHI (median (IQR) 2.0 (0.9–3.8) vs. 19.8 (9.7–34.6), *p* < 0.001) and LAP (median (IQR) 52.8 (31.2–84.3) vs 69.2 (42.1–98.2), p < 0.001) between two groups. And there were no significant differences between non-OSA and OSA in T2DM duration, gender, current smoking, regular drinking, OGLA use, statin use, insulin use, SBP, DBP, TC and LDL-c.

**Table 1:** Demographic, lifestyle and clinical characteristics of study subjects

	Total	None-OSA	OSA	*p* value
n (%)	317	112 (35.3%)	205 (64.7%)	
Age (years)	52.4 ± 14.1	47.1 ± 14.8	55.3 ± 12.8	< 0.001#
Gender				0.357
Female (n, %)	98 (30.9%)	31 (27.7%)	67 (32.7%)	
Male (n, %)	219 (69.1%)	81 (72.3%)	138 (67.3%)	
T2DM duration (years)	4.0 (1.0–10.0)	3.0 (0.6–8.0)	4.0 (1.0–10.0)	0.056
Current smoking (%)	112 (35.3%)	40 (35.7%)	72 (35.1%)	0.916
Regular drinking (%)	100 (31.5%)	33 (29.5%)	67 (32.7%)	0.556
OGLA use (%)	249 (78.5%)	83 (74.1%)	166 (81.0%)	0.154
Stain use (%)	234 (73.8%)	71 (63.3%)	163 (79.5%)	0.001#
Insulin use (%)	208 (65.6%)	76 (67.9%)	132 (64.4%)	0.283
SBP (mmHg)	133 ± 18	131 ± 17	134 ± 18	0.058
DBP (mmHg)	81 ± 10	79 ± 10	81 ± 10	0.071
BMI (kg/m^2^)	27.9 ± 4.1	27.3 ± 4.2	28.2 ± 4.0	0.039*
WC (cm)	98.0 ± 9.9	95.3 ± 9.8	99.5 ± 9.6	< 0.001#
HbA1c (%)	9.9 ± 2.2	10.1 ± 2.3	9.8 ± 2.2	0.160
TG (mmol/L)	1.69 (1.20–2.51)	1.55 (1.15–2.20)	1.76 (1.28–2.58)	0.041*
TC (mmol/L)	5.2 ± 1.1	5.3 ± 1.2	5.1 ± 1.1	0.252
HDL-c (mmol/L)	1.1 ± 0.3	1.1 ± 0.2	1.1 ± 0.2	0.244
LDL-c (mmol/L)	3.1 ± 1.1	3.3 ± 1.1	3.1 ± 1.0	0.048*
AHI (events/h)	9.2 (3.4–25.5)	2.0 (0.9–3.8)	19.8 (9.7–34.6)	< 0.001#
LAP	60.8 (37.2–94.0)	52.8 (31.2–84.3)	69.2 (42.1–98.2)	0.001#

^*^ *p* < 0.05; ^#^ *p* < 0.01

Data was presented as the mean ± standard deviation or as median (interquartile range) for continuous variable or number and percentage for categorical variable

*Abbreviations: T2DM* type 2 diabetes mellitus, *OGLA* oral glucose-lowering agents, *WC* Waist circumference, *BMI* body mass index, *SBP* systolic blood pressure, *DBP* diastolic blood pressure, *TG* Triglyceride, *TC* total cholesterol, *HDL-c* high-density lipoprotein cholesterol, *LDL-c* low-density lipoprotein cholesterol, *AHI* apnea hypopnea index, *OSA* obstructive sleep apnea syndrome, *LAP* Lipid accumulation product

### Correlation of LAP with AHI

**Fig. 1:**
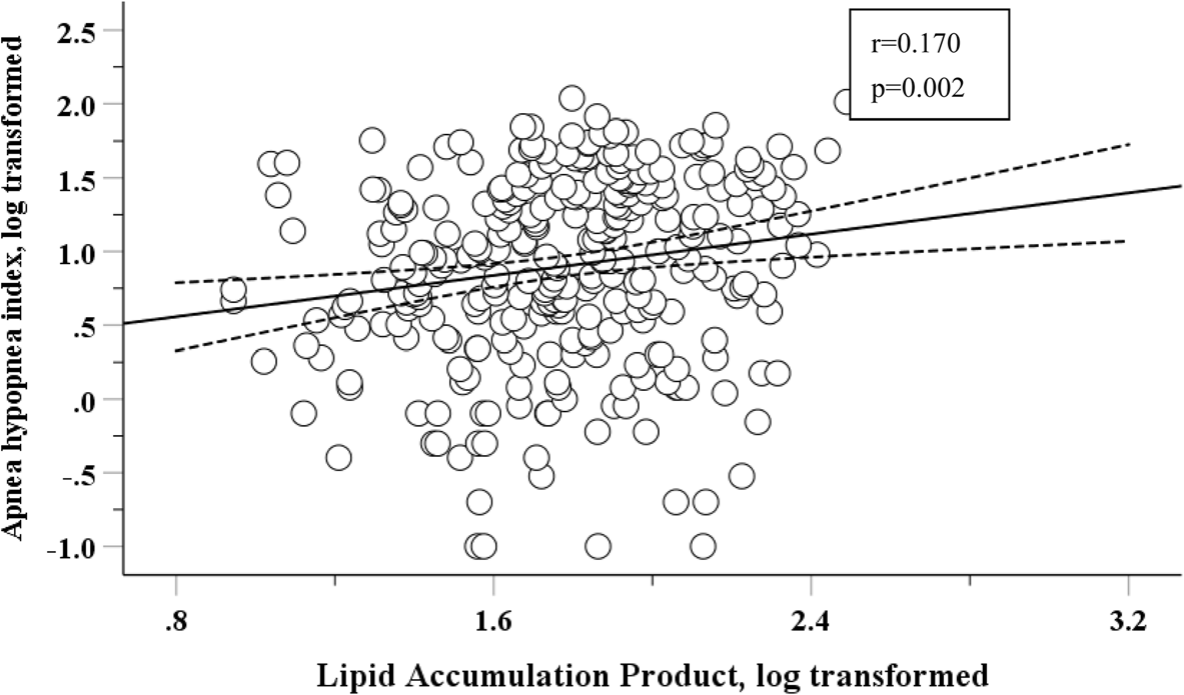
Correlation of LAP (log-transformed) with AHI (log-transformed) in patients with T2DM

Pearson’s correlation analysis was performed to explore the correlation of LAP (log-transformed) with AHI (log-transformed). Figure 1 showed that LAP was significantly and positively correlated with AHI, with the Pearson’s correlation coefficient of 0.170 (*p* = 0.002).

### Association of LAP with AHI

Multivariate linear regression analyses were performed to show the independent association of LAP with AHI (Table 2). In model 1, model 2 and model 3 with adjustment for the same confounding factors as OSA, the adjusted linear regression coefficients (95% CI) of per SD increase of LAP for AHI (log-transformed) were 0.100 (0.032–0.169, *p* = 0.004), 0.121 (0.052–0.191, *p* = 0.001) and 0.092 (− 0.011–0.194, *p* = 0.080), respectively. Similar to logistic regression analyses on OSA, the full models (models 3) showed neither TG nor WC was significantly associated with AHI. Akaike information criterion (AIC) of multivariate linear regression analyses of TG, WC and LAP in model 3 were 543.5, 543.9 and 541.6, respectively.

**Table 2:** Associations of TG, WC and LAP with AHI and OSA in patients with T2DM

	Linear regression on Log (AHI)			Logistic regression on OSA		
	Coefficient	95%CI	P value	ORs	95%CI	P value
**TG**						
**Model 1**	0.029	−0.040 to 0.099	0.406	1.242	0.965 to 1.599	0.092
**Model 2**	0.043	−0.027 to 0.113	0.227	1.336	1.007 to 1.773	0.045
**Model 3**	0.060	−0.041 to 0.160	0.244	1.352	0.876 to 2.085	0.173
**WC**						
**Model 1**	0.019	0.013 to 0.026	< 0.001	1.048	1.021 to 1.075	< 0.001
**Model 2**	0.022	0.016 to 0.029	< 0.001	1.067	1.036 to 1.099	< 0.001
**Model 3**	0.008	−0.004 to 0.019	0.186	1.038	0.989 to 1.090	0.133
LAP						
**Model 1**	0.100	0.032 to 0.169	0.004	1.520	1.159 to 1.995	0.003
**Model 2**	0.121	0.052 to 0.191	0.001	1.752	1.273 to 2.413	0.001
**Model 3**	0.092	−0.011 to 0.194	0.080	1.639	1.032 to 2.604	0.036

AHI was log-transformed

Model 1 was not adjusted

Model 2 was adjusted for age, gender, T2DM duration, regular drinking, current smoking, OGLA, statin use, and insulin use

Model 3 was further adjusted for BMI, SBP, DBP, TC, LDL-c, HbA1c

*Abbreviations: T2DM* type 2 diabetes mellitus, *OGLA* oral glucose-lowering agents, *WC* Waist circumference, *BMI* body mass index, *SBP* systolic blood pressure, *DBP* diastolic blood pressure, *TG* Triglyceride, *TC* total cholesterol, *HDL-c* high-density lipoprotein cholesterol, *LDL-c* low-density lipoprotein cholesterol, *AHI* apnea hypopnea index, *OSA* obstructive sleep apnea syndrome, *LAP* Lipid accumulation product

### Association of LAP with OSA

Multivariate logistic regression analyses were performed to show the independent association of LAP with OSA (Table 2). In model 1 without any adjustment, the adjusted OR (95% CI) of per SD increase of LAP for OSA was1.520 (1.159–1.995, *p* = 0.003). In model 2 and model 3 with the same adjustments as those in multivariable linear regression analyses, the association of LAP with OSA was still statistically significant, with the adjusted OR (95%CI) of per SD increase of LAP of 1.752 (1.273–2.413, *p* = 0.001) and 1.639 (1.032–2.604, *p* = 0.036), respectively. Additional multivariate logistic regression analyses were also performed to show the independent associations of OSA with the two constitutes of LAP (TG and WC). In the full models (models 3) with adjustment for potential confounding factors, neither TG nor WC was significantly associated with OSA; and the adjusted ORs (95%CI) were 1.352 (0.876–2.085) and 1.038 (0.989–1.090) (both *p*-values > 0.05). AIC were 382.0, 381.1 and 379.7 for TG, WC and LAP in model 3, respectively.

**Fig. 2:**
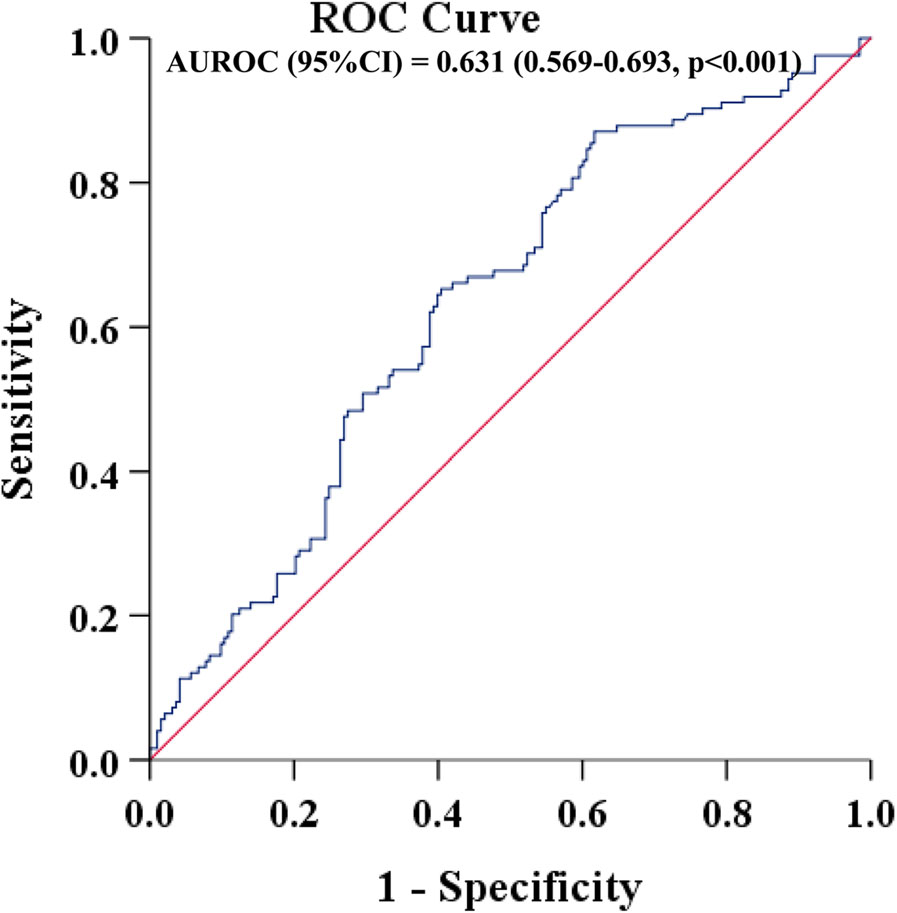
Receiver operating characteristic curves and area under receiver operating characteristic curves (AUROCs) for the detection of moderate-severe OSA using LAP

Further, ROC analysis was used to determine the suggested cutoff values of LAP for moderate-severe OSA. The area under the ROC curve (AUROC) (95%CI) for LAP was 0.631 (0.569–0.693, *p* < 0.001). The best cut-off points of LAP to detect moderate-severe OSA was 40.77 (Youden’s index = 0.254) (Fig. 2).

## Discussion

In the current study, we found that the prevalence rates of OSA were 63.0% for men, and 68.4% for women in this diabetic patient’scohort. LAP was significantly correlated with AHI. Furthermore, LAP was significantly associated with OSA after adjusting for potential confounding factors in both linear regression and logistic regression analyses. However, with adjustment for same confounding factors, as constituents of LAP, neither TG nor WC was significantly associated with AHI and OSA. Our results indicated that LAP was independently associated with the risk of OSA.

There is a bidirectional association between OSA and T2DM [4, 26]. OSA is characterized by the intermittent hypoxemia through repeated upper airway collapse. The repeated episodes of obstructive apnea and hypopnea would lead to metabolic disorders such as insulin resistance, impairment of glucose tolerance and development of T2DM [14], which eventually lead to diabetes and cardiovascular disease [27, 28]. A recent meta-analysis of nine longitudinal cohort studies included 64,101 participants from various geographic regions, with a follow-up duration between 2.7 and 16 years, revealed that OSA is associated with incident diabetes, with an adjusted pooled relative risk of 1.35 [4]. Conversely, individuals with diabetes also increase the risk of developing OSA. In a population-based study including three ongoing prospective U.S. cohorts, the researchers observed a 43% higher OSA risk among individuals with diabetes after adjusting for adiposity [26]. In another study, T2DM have an almost 50% increase in risk of developing OSA compared with patients without T2DM, independent of potential confounders and traditional OSA risk factors [29]. Overweight / obesity is one of important causative factors of both OSA and T2DM, and shared the risk factors for both OSA and T2DM [30].

In terms of AHI and insulin resistance, the severity of OSA is both associated with visceral obesity rather than BMI [31]. Similarly, visceral fat has been associated with increased risk of OSA in those with T2DM [32]. In type 2 diabetic patients with poor diabetic control, OSA is highly prevalent and related to abdominal obesity [33]. A recent cross-sectional study indicated that high value of either WC or waist-to-height ratio is associated with the presence and severity of OSA [34]. Magnetic resonance imaging (MRI) and computed tomography (CT) are the gold standards we know for measuring visceral fat. It is difficult to be widely used because of its high cost [35]. LAP is a simple indicator thatrequires only the serum triglycerides level and an anthropometric factor (WC), which can accurately differentiate between visceral adiposity and subcutaneousadiposity by describing over accumulation of fat mass [36]. Previous studies reveal LAP is often used to predict and identify diseases such as diabetes, metabolism syndrome, polycystic ovary syndrome and CVD [16, 19, 37–39]. A recent large cross-sectional study shows that LAP has a modest effect in screening OSA [21]. BMI is extensively used as the generally marker of global adiposity. However, in the current study after adjusting for potential confounding factors, even BMI, we found that LAP was still independently associated the risk of OSA in patients with T2DM. There may be several potential mechanisms for LAP in association with the risk of OSA. Firstly, comparing with the other surrogates such as TG/HDL-C ratio, visceral adiposity index (VAI), TG, fasting glucose (TyG) index, LAP had better association with insulin-stimulated glucose disposal and higher ability to detect insulin resistance with insulin sensitivity assessed by hyperinsulinemic euglycemic clamp [40]; Secondly, longitudinal trajectories of LAP, which reflect the efficacy of patients’ lipid-lowering treatment and lifestyle improvement, has an independent effect on 5-year T2DM incidence beyond LAP measured at baseline [18].

There are some limitations in the present study. First, this is a cross-sectionalstudyand no causal relationship can be drawn. Further prospectivecohort studies are needed to confirm the precise relationship between LAP and OSA in T2DM patients. Second, the sample size of studywas relatively smalland we might not have sufficient power. Third, the participants of our study come from the hospitalized patients with poor controlled glycemia, which cannot represent the general population.

## Conclusion

In conclusion, we found that LAP, a combination index of waist circumference and triglycerides, is independently associated with OSA, even with adjustment for BMI and other potential confounding factors, and therefore could be used as a potential OSA risk marker in T2DM patients, beyond the general index of obesity.

## Data Availability

The datasets used and / or analyzed during the current study are available from the corresponding author on reasonable request.
